# The Effect of the Hemochromatosis (*HFE*) Genotype on Lead Load and Iron Metabolism among Lead Smelter Workers

**DOI:** 10.1371/journal.pone.0101537

**Published:** 2014-07-02

**Authors:** Guangqin Fan, Guihua Du, Huijun Li, Fen Lin, Ziyong Sun, Wei Yang, Chang Feng, Gaochun Zhu, Yanshu Li, Ying Chen, Huan Jiao, Fankun Zhou

**Affiliations:** 1 Department of Occupational Health and Toxicology, School of Public Health, Nanchang University, Nanchang, People's Republic of China; 2 Department of clinical laboratory, Tongji Hospital, Tongji Medical College, Huazhong University of Science and Technology, Wuhan, People's Republic of China; 3 Department of Preventive Medicine, School of Medicine, Gannan Medicine University, Ganzhou, People's Republic of China; 4 Nevada Center for Health Statistics and Informatics, School of Community Health Sciences, University of Nevada, Reno, United States of America; CINVESTAV-IPN, Mexico

## Abstract

**Background:**

Both an excess of toxic lead (Pb) and an essential iron disorder have been implicated in many diseases and public health problems. Iron metabolism genes, such as the hemochromatosis (*HFE*) gene, have been reported to be modifiers for lead absorption and storage. However, the *HFE* gene studies among the Asian population with occupationally high lead exposure are lacking.

**Objectives:**

To explore the modifying effects of the *HFE* genotype (wild-type, *H63D* variant and *C282Y* variant) on the Pb load and iron metabolism among Asian Pb-workers with high occupational exposure.

**Methods:**

Seven hundred and seventy-one employees from a lead smelter manufacturing company were tested to determine their Pb intoxication parameters, iron metabolic indexes and identify the *HFE* genotype. Descriptive and multivariate analyses were conducted.

**Results:**

Forty-five *H63D* variant carriers and no *C282Y* variant carrier were found among the 771 subjects. Compared with subjects with the wild-type genotype, *H63D* variant carriers had higher blood lead levels, even after controlling for factors such as age, sex, marriage, education, smoking and lead exposure levels. Multivariate analyses also showed that the *H63D* genotype modifies the associations between the blood lead levels and the body iron burden/transferrin.

**Conclusions:**

No *C282Y* variant was found in this Asian population. The *H63D* genotype modified the association between the lead and iron metabolism such that increased blood lead is associated with a higher body iron content or a lower transferrin in the *H63D* variant. It is indicated that *H63D* variant carriers may be a potentially highly vulnerable sub-population if they are exposed to high lead levels occupationally.

## Introduction

There is considerable variability in the toxic response to lead (Pb) exposure among both the general population and occupational workers. For exposed persons, the outcome may be affected by factors such as the degree of exposure, age, and genetically determined differences in uptake, elimination, or sensitivity to the toxic effects of the substance. Genetic variants have occurred in enzymes known to influence or regulate lead metabolism. A potential candidate gene for susceptibility to lead exposure is the hemochromatosis (*HFE*) gene encoding the *HFE* protein. The presence of mutations in the *HFE* gene (major in *C282Y* variant and less common in *H63D* variant) leads to hemochromatosis. Hemochromatosis is an autosomal recessive hereditary disease in which an abnormality in iron metabolism, consisting in increased iron absorption by the gut and increased export of iron by the reticulum-endothelial system, leads to iron accumulation and deposits in multiple internal organs. The result is the progressive tissue damage as fibrosis/cirrhosis, diabetes, arthropathy, cardiomiopathy, hipopituitarism among others. The *HFE* gene is localized in chromosome 6p21.3 and two predominant variants have been described, the *C282Y* variant and the *H63D* variant and the *C282Y* variant is associated in homozygous in more than 80% with Hereditary Hemochromatosis [1].

Pb is non-essential to humans. Therefore, it is unlikely that a specific transporter exists for it. Pb shares the same pathway as Fe because they are both divalent cations, and our previous study has shown that Pb and Fe may share the same transporters, such as the divalent metal transporter 1 (DMT1) and ferroportin 1 (FP1) [2]. Iron status may over- or under-regulate intestinal divalent cation transporters and has been suggested specifically with the *HFE* genotype [3], [4]. Because of the associations between iron and lead transport, it is possible that polymorphisms in the *HFE* gene may also influence lead absorption and storage. Studies have shown mixed results.

Considering the clinical association between Fe deficiency and Pb toxicity, several studies have shown that the *HFE* mutations (*H63D* variant and *C282Y* variant) were significantly associated with increased blood lead (BPb) levels in young children [5]. However, in a cohort study of elderly men, *HFE* mutation predicted lower patella and BPb levels [4]. Additionally, a study of 2,926 Australian adult male and female twins showed that the *HFE* genotype did not contribute to variations in the BPb levels in adults [6]. Hopkins et al. [5] proposed that the *HFE* gene affects the BPb levels, which vary by age because of the differences in iron needs and storage that correlate with age and sex. Furthermore, they proposed that in younger populations, which have higher iron needs, *HFE* mutation may increase Pb absorption among the variant's carriers. However, the results did not demonstrate evidence of the BPb levels' modification by serum ferritin or hemoglobin in children or adults. Conversely, in an older population of elderly males, the effect of the *HFE* mutation may contribute to the down-regulation of Fe and Pb absorption. However, this study lacked data on the analysis concerning iron storage. Iron status is plausibly related to BPb levels and *HFE* genotype and should theoretically be a modifier of the association of *HFE* with BPb rather than a confounder. The conclusions of three previous studies differ from each other, suggesting that gene's environmental interactions may vary by life stage and Pb exposure.

The existing research about the association between *HFE* genotype and Pb exposure was performed on the general population and cannot directly illuminate the causes of variation in more highly exposed groups of Pb workers. The Meta analyses of the published data show that individuals with ALAD polymorphism have higher BPb levels at high Pb exposures, whereas there is no difference at low Pb exposures [7], [8], [9]. Moreover, owing to the wide variability in the prevalence rates of *HFE* mutation, such as *C282Y*, in particular the rare reports of the *C282Y* variant in China, it will be important to replicate the study of these genetic associations in different populations.

In the present study, we measured the biomarkers associated with the exposure, distribution, and excretion of Pb [BPb and urine lead (UPb)] and the body iron metabolic indexes in Chinese Pb-workers. In this study, we used unexposed factory workers as controls. In particular, we examined the distribution of the *HFE* genotype and the potential genetic modifications of the biomarkers of toxicokinetics and their associations with toxic effects. The analysis includes a consideration of covariates such as sex, age, smoking, alcohol intake, and education level to better understand the effects of the *HFE* genotype on the association between Pb exposure and Fe metabolism.

## Materials and Methods

### Studied Populations

Eight hundred and thirty-one individuals were identified from a lead smelter. Those who were exposed to lead smoke/lead dust directly, such as through lead extractive metallurgy or electrolytic lead, were recruited into the exposed group. Other individuals who worked in the offices distant from the workplaces associated with lead exposure were recruited into the control group, and the controls were workers of the same lead smelter but unexposed. Subjects who did not complete a questionnaire, failed to provide biological samples, or were diagnosed with a disorder of the peripheral nervous system, diabetes mellitus, limb injuries and/or drug intoxication were excluded. Seven hundred and seventy-one individuals were finally recruited for the study, of which 705 were lead-exposed individuals (576 males and 129 females, with a median employment of 10.63 years) and 66 controls (43 males and 23 females). The survey was approved by The Clinical Ethics Committee of First Affiliated Hospital of Nanchang University. Written informed consent was obtained from each individual prior to the study.

A standardized questionnaire was used to elicit their ethnic backgrounds, alcohol and smoking habits (yes/no), protective measures, self-reported symptoms, medical history and occupational history, such as work unit (department), type of work, and exposure duration.

### Sample Collection

All of the samples were obtained from the participants during a fasting state at the same time of the morning. Blood samples were obtained by venipuncture after overnight (>12 h) fasting: 2 ml of blood was collected in a vacutainer with K_2_EDTA anticoagulant for extracting the DNA of the lymphocytes, and 1 ml was collected in a plastic “metal-free” evacuated tube containing sodium heparin anticoagulant for determining the BPb and zinc protoporphyrin (ZPP) immediately. At the same time, 2 ml of blood was collected in a tube with a non-anticoagulant for extracting serum samples. Spot urine samples were collected in acid-washed polyethylene bottles. Each urine sample was acidified with concentrated nitric acid and was used for Pb analysis. All of the samples except for the blood samples used to determine BPb and ZPP were frozen at −20°C until the survey was finished (2–3 days) and then stored at −80°C until the beginning of the analysis.

### Evaluation of Lead Intoxication Parameters

BPb and UPb were measured by a stripping out analyzer (MP-2, Shandong, China), using the differential potentiometer stripping method (WS/T 21-1996) and the oscillo-polarographic method (WS/T 91-1996), respectively. A quality control sample was inserted in each run of the 10 samples. The limits of detection for Pb were 0.9 µg/L in blood and 5 µg/L in urine, and the precision was 6.1% and 5.4%, respectively. The accuracy was assured with certified blood and urine samples from the National Center for Disease Control and Prevention of China; 303±5.8 µg/L in blood and 260±6.5 µg/L in urine (recommended 299±11 µg/L and 252±30 µg/L).

The ZPP concentration was assayed directly by a ZPP blood fluorescence determinator (model 2002, Xian, China), using the hematofluorometer method (WS/T 92-1996), which measured the ratio of the fluorescence of ZPP to the absorption of the light by sample (by hemoglobin (Hb)) and is presented as the µg ZPP/g of the Hb (µg/g Hb). The blood concentration of Hb was assayed by the cyanomethemoglobin colorimetric assay method.

### Measurement of Serum Iron Indices

Serum samples were obtained in the fasting state at the same time of day, stored at −20°C, and then sent to the Clinical Laboratory Department of Tongji Hospital (Wuhan, China) together. The spectrophotometric determination of serum iron (sFe, reference interval, 10.6–28.3 µmol/L for men and 6.6–26.0 µmol/L for women) and unsaturated iron-binding capacity (UIBC, reference interval, 7.5–65.3 µmol/L) were measured using the Roche (Modular P) automatic biochemical analyzer (Roche Diagnostics, Mannheim, German). The turbidimetric immunoassay of the serum ferritin (sFn, reference interval, 20–290 µg/L for men and 4.5–170 µg/L for premenopausal women, 24–260 µg/L for postmenopausal women), transferrin (Tf, reference interval, 2.0–3.6 g/L) and soluble transferrin receptor (sTfR, reference interval, 0.76–1.76 mg/L) were determined using GmbH (Marburg, Germany). The total iron binding capacity (TIBC) was calculated from these results as follows: TIBC = Fe+UIBC. The serum transferrin saturation (TfS) was calculated based on the formula TfS (%) = Fe/TIBC×100. The body iron content was calculated based on the formula (BIC, mg/kg) = −[log(sTfR/sFn ratio)-2.8229]/0.1207.

The CVs for inter-day precision near the upper bounds of the reference intervals for ferritin, Tf and sTfR were 3.1%, 2.6%, and 1.2%, respectively. The inter-day CV was 1.2% for sFe and 1.6% for UIBC, with means of 9.31 µmol/L and 32.9 µmol/L, respectively.

### Identification of the *H63D* and *C282Y* Variant in the Hemochromatosis Gene

The genomic DNA was extracted from the subjects' peripheral white blood cells using the improved NaI method [10]. Briefly, this method involves the treatment of whole blood with an equal volume of NaI [3 M final concentration, analytical grade, purchased from Bodi Chemical Reagent Co., Ltd (Tianjing, China)] followed by a chloroform-isoamyl alcohol extraction to clear the Hb and cell debris. The clear aqueous layer is then mixed with isopropanol to obtain the DNA. Finally, the DNA was dissolved in a TE buffer (containing Tris, a common pH buffer, and EDTA, a chelating agent). After the DNA quantification, the samples were adjusted to the TE buffer, partitioned into aliquots, and stored at −20°C.

The *C282Y* (rs1800562) and *H63D* (rs1799945) variants (Reference Sequence NM 139011, GenBank) were genotyped by a PCR and a restriction fragment length polymorphism (RFLP) analysis as previously described [11]. Briefly, two portions of the *HFE* gene surrounding both mutations were amplified separately using PCR with *HFE*-specific primers. These primers was designed using Primer Express v5.0 software (Applied Biosystem) based on the published sequence of the *HFE* gene and synthesized from Sangon Biotech Co., Ltd (Shanghai, China). The PCR reactions consisted of 20 µM of each primer, forward PCR primer, 5′- TGT GGA GCC TCA ACA TCCT-3′; reverse PCR primer, 5′- TGA AAA GCT CTG ACA ACC TCA-3′ for the *H63D* variant (rs1799945), and forward PCR primer, 5′ -TCC AGT CTT CCT GGC AA-3′; reverse PCR primer, 5′-TTC TAG CTC CTG GCT CTCA-3′ for the *C282Y* variant (rs1800562), 2.5 µl 10×Reaction Buffer (containing Mg^2+^), 2 µl dNTP (2.5 mM), 0.125 µl Ex Taq DNA polymerase(5 U/µl), and 4 µl genomic DNA; then sterile water was added to 25 µl of Ex Taq DNA polymerase, 10×Reaction Buffer, dNTP (100 mM) and 6×Loading Buffer, which were purchased from Takara (Dalian, China). The PCR conditions were 3 min at 94°C, followed by 35 cycles of 30 sec at 94°C, 30 sec at 56°C and 1 min at 72°C, and finally, 5 min at 72°C, performed using the autorisierter thermocycler (eppendorf Mastercycler personal, Germany).

After the PCR amplification, restriction digests were performed directly with 10 µl of the PCR mixtures by the addition of 5 U SnaBI for *C282Y* (BioLabs, New England, MA, USA) or BclI (BioLabs) for *H63D* and the corresponding buffers (2 µl 10×NEB4 Buffer added to 2 µl 10×BSA, 5 µl sterile water and 2 µl 10×NEB3 Buffer and adding sterile water to reach 20 µl) and incubated for 2 h at 37°C for the SnaB I restriction or overnight at 50°C for the Bcl I restriction. The products were resolved on 2% agarose gel and stained by ethidium bromide (EB). A random sample of 10% of the subjects was run in duplicate as a quality control measure. The genotypes were also determined in control blood known to be from subjects homozygous for the wild-type genotype and heterozygous and homozygous for each of the variant genotypes.

### Statistical Methods

We conducted a cross-sectional analysis of the association between the *HFE* mutation and the BPb/UPb concentrations and the iron metabolic indexes among Pb-exposed and unexposed workers in a Pb smelter. We first compared the characteristics of subjects who had all of the data of interest, including the genotype, BPb and UPb levels, and the iron metabolic index with the subjects who were not included because of missing data. The allele and genotype frequencies were determined and the Hardy-Weinberg test for equilibrium was performed. The univariate distributions of the continuous variables were examined to determine departures from normality. The distributions of the demographic and lifestyle characteristics and the BPb/UPb/ZPP levels and iron metabolic index by genotype (wild-type vs. *C282Y* variant or *H63D* variant) were examined, and the differences were tested by the Chi-square test, Student's t-test or the nonparametric test, as appropriate. The distribution of ZPP evidenced departures from normality and was thus transformed to a natural logarithm (ln) in the hypothesis tests between the groups of exposed and non-exposed workers. The adequacy of the ln-transformation of these measures was confirmed by verification that the distributions of the residuals after the linear regression modeling were normal.

Multiple linear regressions were used to model the effect of the genotype on the Pb burden and the iron metabolic index in cross-sectional analyses, while controlling for the same covariates used in the final models. Cross-product terms for the genotype and Pb burden variables or for the genotype and the iron metabolic indexes were used to evaluate the effect of the modification by genotype on the association between Pb exposure and body iron status. In consideration of the correlation among the iron metabolic indexes (sFe, UIBC, sFn, Tf, sTfR, TIBC, TfS, BIC, Hb), these variables were used separately in the final regression models to predict the Pb burden (BPb/UPb/ZPP), and each of these regressions was repeated, adding an indicator variable for the genotype. Covariates for the final regression model were identified by stepwise forward modeling (based on the *P* value) of the age, gender (male vs. female), education (lower than high school vs. higher than high school), tobacco use (yes vs. no), alcohol consumption (yes vs. no), occupational Pb exposure (unexposed, dissolved Pb operations or electrolytic Pb operations) and exposed/unexposed work duration.

The statistical analysis was performed using SPSS 19.0 software. Statistical significance was considered to be present at a *P<0.05*.

## Results

The lead biomarkers BPb, UPb, ZPP of the exposed workers were 364.90±150.83 µg/L, 36.00 (17.00, 75.00) µg/L and 6.10 (3.40, 10.85) µg/g Hb, respectively, and were significantly higher than those of the unexposed workers (142.99±58.62 µg/L, 2.50 (1.00, 7.00) µg/L and 0.90 (0.00, 1.95) µg/g Hb), respectively. The means and the distributions of the iron metabolism indexes (sFe, TfS, sFn, BIC) and education, drinking, and the years that the males were employed were different between the exposed and unexposed workers. However, the distributions of smoking and the *HFE* genotypes, as well as the means of UIBC, Tf, TIBC, sTfR, and Hb, among all of the subjects were similar (Table 1, 2). The overall prevalence values for the *H63D* genotypes were wild-type, 94.16% (726/771); heterozygous, 5.71% (44/771), with 5.39% (38/705) for exposed group and 9.09% (6/66) for unexposed group; and homozygous, 0.13% (1/771), with 0.14% (1/705) for exposed group. Allelic frequency in global was 2.98% (46/1542) in this population, with 2.84% (40/1410) for exposed workers and 4.55% (6/132) for unexposed workers. The prevalence values of the *C282Y* genotypes were wild-type, 100%; heterozygous and homozygous, 0%. The distribution of the *H63D* genotype conformed to the Hardy-Weinberg expected frequencies (*H63D*: Chi-square = 0.011, *P = 0.995*).

**Table 1:** Demographic Characteristics of Subjects by Exposure [M(P_25_, P_75_)or N(%)].

Grouping	Exposed		Unexposed	
	Males	Females	Males	Females
Number	576(81.7)**	129(18.3)**	43(65.2)	23(34.8)
Age (years)	32.0(23.0, 39.0)	38.0(34.0, 41.0)**	26.5(24.0, 41.5)	26.0(23.0, 39.0)
Exposed/unexposed work duration (years) exposure	2.6(1.6, 18.2)	18.0(12.0, 21.0)*	2.0(1.6, 2.8)	2.6(2.4, 21.0)
Education (≥high school)	79(14.3)**	11(8.8)**	28(68.3)	15(65.2)
Married	377(67.2)	109(87.2)**	23(54.8)	14(60.9)
Smoker	351(62.9)	0(0.0)	24(58.5)	0(0.0)
Drinker	320(57.7)*	1(0.8)	31(75.6)	0(0.0)

*(**) *p<0.05* (*0.01*) (Chi-square test or nonparametric test) compared with the unexposed group of the same sex.

Results were expressed as the mean ± SD when the continuous variables followed a normal distribution.

Results were expressed as M (P25, P75) when the continuous variables did not follow a normal distribution.

**Table 2:** Lead Biomarkers, Serum Iron Indices and Genotypes by Exposure [Mean ± SD or N (%) or M (P_25_, P_75_)].

Grouping	Exposed		Unexposed	
	Males	Females	Males	Females
BPb(µg/L)	384.54±151.98**	276.16±107.93**	144.72±60.22	139.91±56.84
UPb(µg/L)	39.00(18.00,80.00)**	28.50(12.00,51.50)**	3.00(1.00,7.00)	1.00(1.00,7.50)
ZPP(µg/gHb)	9.00±8.92**	9.03±8.24**	2.49±1.24	4.98±5.79
sFe (µmol/L)	20.97±7.90*	17.08±7.13	24.00±7.93	14.74±7.00
UIBC(µmol/L)	37.4±12.4	44.2±13.9*	35.4±12.7	51.1±14.8
TIBC(µmol/L)	56.90(51.79, 62.73)	61.3±10.8	57.83(52.65, 65.29)	65.9±12.1
Tf (g/L)	2.73±0.49	2.92±0.54	2.87±0.42	2.96±0.58
TfS (%)	34.80(26.10, 44.96)*	28.92±12.84	41.37(30.31, 49.68)	23.39±11.65
sFn (µg/L)	160.00(92.30, 230.00)**	38.90(20.95, 59.00)	238.00(148.00, 343.00)	42.20(20.70, 64.50)
sTfR (mg/L)	1.30(1.13, 1.55)	1.38(1.20, 1.56)*	1.36(1.12, 1.55)	1.26(0.99, 1.44)
BIC (mg/kg)	40.30±2.63**	35.22±3.28	41.74±2.67	35.46±2.69
Hb(g/L)	139.8±15.0	120.5±12.2	138.3±13.3	115.9±14.2
*H63D*				
HH	546(94.8)	120(93.0)	39(90.7)	21(91.3)
HD	29(5.0)	9(7.0)	4(9.3)	2(8.7)
DD	1(0.2)	0	0	0
*C282Y*				
CC	576(100)	129(100)	43(100)	23(100)
CY	0	0	0	0
YY	0	0	0	0

Abbreviations: BPb, blood lead; UPb, urine lead; ZPP, zinc protoporphyrin; sFe, serum iron; UIBC, unsaturated iron-binding capacity; TIBC, total iron binding capacity; Tf, transferrin; TfS, serum transferrin saturation; sFn, serum ferritin; sTfR, soluble transferrin receptor; BIC, body iron content; Hb, haemoglobin. For *H63D* genotype, HH, wild-type; HD, *H63D* heterozygous variant; DD, *H63D* homozygous variant. For *C282Y* genotype, CC, wild-type; CY, *C282Y* heterozygous variant; YY, *C282Y* homozygous variant.

*(**) *p<0.05* (*0.01*) (Chi-square test, Student's *t*-test or nonparametric test) compared with the unexposed group of the same sex.

Results were expressed as the mean ± SD when continuous variables followed a normal distribution.

Results were expressed as M (P_25_, P_75_) when continuous variables did not follow a normal distribution.

Table 3 shows the distribution of the BPb, UPb and covariates stratified by genotype. The overall trend of the lead in the body levels was for the blood levels in the carriers of the *H63D* variant to be slightly higher, but there was no statistically significant difference compared with the wild-type subjects. The UPb/ZPP levels for the carriers of the *H63D* variant did not differ from those of the wild-type subjects. The covariates, except for UIBC, TIBC, and Tf, were similar between the subjects with one copy of the allele and the wild-type.

**Table 3:** Lead Biomarkers and Serum Iron Indices by *HFE* Genotypes [Mean ±SD or N or M (P_25_, P_75_)].

	All		HH		HD or DD		
	N^a^	Mean ±SD or M (P_25_, P_75_)	N	Mean ±SD or M (P_25_, P_75_)	N	Mean ±SD or M (P_25_, P_75_)	*P*
BPb (µg/L)	748	345.90±157.95	705	344.75±158.70	43	364.87±145.52	0.418
UPb (µg/L)	727	32.00(12.300,71.00)	686	31.00(12.00,71.00)	41	40.00(18.00,77.00)	0.536
ZPP(µg/gHb)	757	5.60(3.00,10.20)	714	5.70(3.00,10.33)	43	4.90(2.80,9.40)	0.406
sFe (µmol/L)	769	20.30±7.98	724	20.27±7.96	45	20.72±8.31	0.728
UIBC(µmol/L)	768	37.45(30.60,46.38)	723	37.80(30.90,46.80)	45	33.40(26.15, 42.25)	0.012*
TIBC (µmol/L)	768	57.43(52.40,63.87)	723	57.90(52.55,64.18)	45	54.85(49.11, 60.05)	0.008**
Tf (g/L)	768	2.72(2.44,3.04)	723	2.74(2.46,3.05)	45	2.54(2.23, 2.85)	0.005**
TfS (%)	768	33.41(24.47,44.09)	723	33.13(24.41,43.57)	45	37.19(25.47, 47.17)	0.195
sFn (µg/L)	768	129.00(66.08,214.00)	723	129.00(65.80,214.00)	45	130.00(66.95, 227.00)	0.983
sTfR(mg/L)	755	1.31(1.14,1.55)	710	1.31(1.14,1.55)	45	1.34(1.15, 1.54)	0.844
BIC(mg/kg)	753	39.91(37.53,41.71)	708	39.90(37.49,41.75)	45	40.03(37.77, 41.64)	0.853
Hb (g/L)	762	135.8±16.3	717	135.7±16.3	45	137.0±15.8	0.606

Abbreviations: BPb, blood lead; UPb, urine lead; ZPP, zinc protoporphyrin; sFe, serum iron; UIBC, unsaturated iron-binding capacity; TIBC, total iron binding capacity; Tf, transferrin; TfS, serum transferrin saturation; sFn, serum ferritin; sTfR, soluble transferrin receptor; BIC, body iron content; Hb, haemoglobin; HH, wild-type; HD, *H63D* heterozygous variant; DD, *H63D* homozygous variant.

*(**) *p<0.05* (*0.01*) compared with *HH* (student's *t*-test or nonparametric test).

a Because of missing data, the numbers do not equal 771.

Results were expressed as the mean ± SD when continuous variables followed a normal distribution.

Results were expressed as M (P_25_, P_75_) when continuous variables did not follow a normal distribution.

However, the above analysis does not take into account several potential confounders. Some factors in distribution were unbalanced between the group of Pb-exposed and of unexposed, such as gender, educational levels and iron metabolism indexes et al. From Table 2, Pb-exposed women had lower BPb and UPb values than men, as well as lower sFn and Hb values. Thus, those factors were considered in the multivariate analyses.

Multiple regression models for evaluating the effect of the *H63D* genotype on the association between Pb exposure and iron metabolism are presented in Table 4. In the multiple regression models, no evidence was found that the *H63D* variants directly influenced either the Pb exposure or the iron metabolism. However, the *H63D* genotype did modify associations between the BPb levels, and BIC/Tf was observed. Per unit joint change in BIC with wild-type, there was an −1.149 unit impact to BPb levels (*P = −0.008*), while per unit joint change in BPb with wild-type, there was an −0.002 unit impact to BIC levels (*P<0.001*) (the first and second analysis in Table 4).

**Table 4:** Linear regression models evaluating effect of *HFE* genotypes on the association between lead exposure and iron metabolism.

	*β*	SE	*Bcoff*	*t* value	*P*-value^1^	*R* 2	*F value*	*P-*value^2^
Variable				BPb(µg/L)				
Content	340.588	65.512		5.199	0.000			
BIC	−5.507	1.953	−0.119	−2.280	0.005			
BIC×HH or HD DD	−1.149	0.432	−0.099	−2.661	0.008	0.594	135.187	0.000
				BIC(mg/kg)				
Content	40.661	0.532		76.374	0.000			
BPb×HH or HD DD	−0.002	0.000	−0.284	−9.254	0.000	0.444	135.408	0.000
				Tf(g/L)				
Content	3.649	0.109		33.474	0.000			
BPb	−0.002	0.001	−0.538	−2.877	0.004			
BPb×HH or HD DD	0.000	0.000	0.414	2.237	0.026	0.141	26.626	0.000

Abbreviations: BPb, blood lead; Tf, transferrin; BIC, body iron content; HH, wild-type; HD, *H63D* heterozygous variant; DD, *H63D* homozygous variant.

Linear models were adjusted stepwise for age (year), gender (male vs. female), education (lower than high school vs. higher than high school), marriage (yes vs. no), tobacco use (yes vs. no), alcohol consumption (yes vs. no), occupational lead exposure (unexposed, dissolved lead operations or electrolytic lead operations) and work years. *H63D* genotype (HH vs. HD or DD), iron metabolic index/BPb and the cross-product with the genotype and each iron metabolic index/BPb. While BPb was independent factor, each iron metabolic index and the cross-product with the genotype were put separately into the model.

1 *P* value for each statistic;

2 *P* value for each regression model.

**Figure 1:**
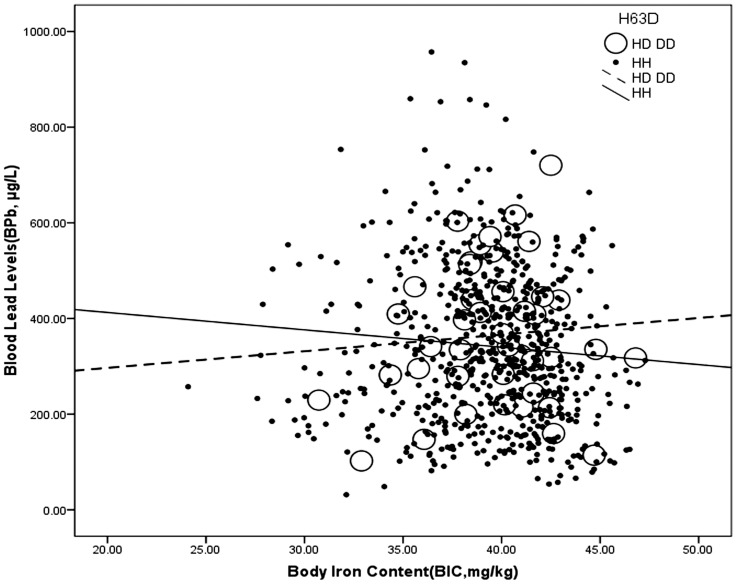
Interactive effect between the *H63D* genotype and body iron content on blood lead levels. *H63D* genotype: HD or DD: open dots, dashed line; HH: close dots, full line. The data are analyzed using multivariate analysis.

The visual inspection of polymorphism was performed on the plots depicting associations between the BPb and BIC or the BPb and Tf stratified by genotype for evidence of a dominant or recessive effect of an allele. The plot depictions also revealed that there was an interactive effect between the *H63D* genotype and the BIC on the BPb levels. Open dots and dashed line regression line (BPb = 227.201+3.478*BIC) denote *H63D* variant carriers, which means a higher BIC was predicted to mean higher BPb levels, whereas close dots and full line regression line (BPb = 485.247–3.634*BIC) denote workers with wild-type, a higher BIC was predicted to lower the BPb levels (Fig. 1).

**Figure 2:**
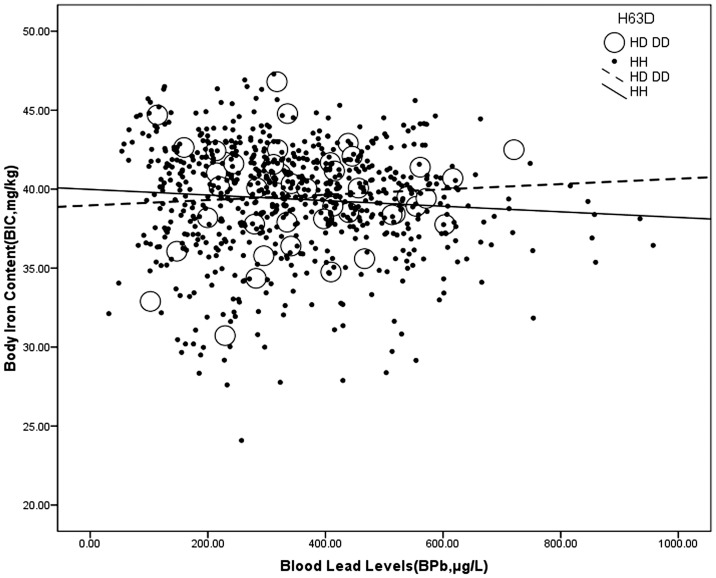
Interactive effect between the *H63D* genotype and blood lead levels on body iron content. *H63D* genotype: HD or DD: open dots, dashed line; HH: close dots, full line. The data are analyzed using multivariate analysis.

Interestingly, the evidence showed that there was an interactive effect between the *H63D* genotype and environmental exposure on the BIC, which revealed that higher BPb levels were associated with a higher BIC in the carriers of the *H63D* variant (open dots and dashed line regression line, BIC = 38.973+0.002*BPb), whereas higher BPb levels were associated with a lower BIC in those without the *H63D* variant (close dots and full line regression line regression line, BIC = 39.984–0.002*BPb) (Fig. 2).

**Figure 3:**
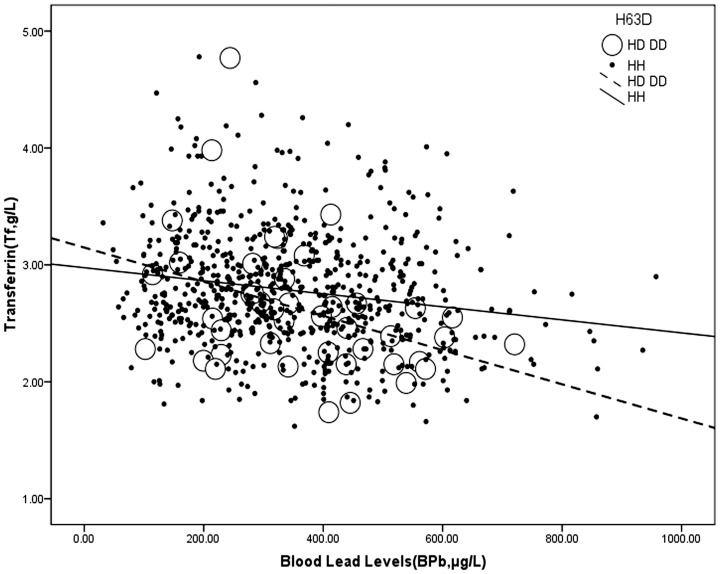
Interactive effect between the *H63D* genotype and blood lead levels on transferrin. *H63D* genotype: HD or DD: open dots, dashed line; HH: close dots, full line. The data are analyzed using multivariate analysis.

Additionally, our results revealed that higher BPb levels were associated with a lower Tf, and a steeper slope was observed in workers with the *H63D* variant (Table 4 and Fig. 3).

The data also revealed that BIC is an independent predictor of significantly lower BPb levels (*βcoff = −0.199, P = 0.005*) in the final regression models that were adjusted for age, sex, smoking, drinking, education, marriage and lead exposure (Table 4).

We also compared the results of the effect modification by genotype on the associations between the iron metabolic index and the same outcomes (UPb and ZPP). No evidence of effect modification by genotype on associations between the iron metabolic index and UPb or ZPP was found.

## Discussion

In our current study of Chinese Pb-exposed workers, we determined the effect of the *HFE* genotype on the relationship between Pb exposure and iron metabolism. First, the effect modification by genotype on association between BPb and BIC was observed. A higher BIC predicted higher BPb levels in the workers with the *H63D* variant, whereas a higher BIC predicted lower BPb levels in the workers without the *H63D* variant. The results also showed the converse to be true, that higher BPb levels were associated with a higher BIC in the workers with the *H63D* variant, while higher BPb levels were associated with a lower BIC in the subjects with the wild-type. Second, in contrast with BIC, higher BPb levels were associated with a lower Tf in the workers with the *H63D* variant. Our results suggest that the *H63D* genotype modifies the association between iron and lead in Chinese lead workers with high occupational exposure, independent of the presence of the *C282Y* allele, although the *H63D* variant has been regarded as nonfunctional for iron overload in Europe. Moreover, it is more likely for workers with the *H63D* variant to have excess iron stores, and the Pb absorption may promote lead poisoning and increase the AD risk.

To the best of our knowledge, this is the first study to examine the association between iron metabolism and lead exposure modified by the *HFE* gene in Asian workers who are occupationally exposed to Pb. The wide age range of 18 to 55 years has allowed us to observe the associations between Pb exposure and iron metabolism at almost every stratified age in adults. Pb shares the same pathway as Fe, as they are both divalent cations, and the iron status may up- or down-regulate the intestinal divalent cation transporters [12], [13]. Numerous studies have shown that the regulatory mechanisms that cause an increase in iron absorption accompany an increase in the amount of ingested lead that is absorbed during iron deficiency [14], [15], [16], [17]. Body iron status is inversely associated with the Pb burden because elevations in the BIC prevent the body from controlling iron's absorption to prevent its systemic excess in humans, and the Pb absorption is subsequently lower [18], [19]. Polymorphisms of the *HFE* gene are of interest in the studies of human Pb exposure because the iron metabolism impacts the Pb absorption from the gastrointestinal tract and the Pb transfer and/or storage. One research group found, based on this hypothesis, that the *HFE* mutation modifies lead metabolism in children and elderly men, revealing a higher blood lead in children and lower blood/bone lead in elderly men carrying the *HFE* mutation. They explained that the *HFE* gene's effect on the blood lead may vary by life stage because of the differences in body iron needs and storages that correlate with age and sex [4], [5]. The conclusions of these previous studies differ from each other and also from our results.

In contrast with other studies, the *C282Y* variant was not detected, whereas 5.8% of the Chinese lead workers in our study carried the *H63D* variant. Although there is no clear data establishing whether the *H63D* variant leads to the phenotypic manifestations of hemochromatosis or significant iron overload, some studies have suggested that the *H63D* variant is associated with a milder form of iron overload or hereditary hemochromatosis [20], [21]. In addition, evidence has been reported in a previous functional study that the *H63D* variant alters the normal *HFE* product's affinity for its ligand [22], which leads to relatively small increments of cellular iron content [23]. An increased hepatic iron deposition was observed in the *H63D* heterozygous variant carriers (with 3 compound heterozygous *C282Y*/*H63D* in 58 *H63D* heterozygous variant) [24]. Iron absorption often accompanies an increase in lead absorption [25]. A case-control study of hereditary hemochromatosis indicated an up-regulation of iron absorption with a subsequent increase in lead absorption [3]. Our multivariate analysis results revealed that BIC was positively associated with BPb levels in individuals with the *H63D* variant, and higher lead values were found in the individuals with a higher body iron content, whereas the reverse occurred in the workers without the *H63D* variant.

Our study differs from previous cohort-based studies assessing lead exposure among the general population when the major *HFE* mutation was *C282Y* variant because we assess high-level occupational lead exposure and focus on Chinese subjects that are *H63D* heterozygous variant for the *HFE* mutation. All of these factors may play a role in explaining the differing results. In a study by Wright et al. [4], iron status was related to blood lead and the *H63D* genotype, but unfortunately, the study lacked data on the iron status, and there were no effect modifications by either serum ferritin or hemoglobin in the Hopkins et al. [5] study. In a general population-based cohort study, Whitfild et al. [6] found no significant associations between the *H63D* genotype and blood lead in adults, and blood lead levels were inversely associated with ferritin. Using serum ferritin or hemoglobin concentrations to estimate body iron has certain limitations, the most important of which is the influence of inflammation or liver disease on the serum ferritin level independent of the body iron stores; however, the sTfR can still be used to detect concurrent iron deficiencies in persons with inflammation. At the same time, quantitative estimates of the body iron content calculated from the R/F ratio greatly enhance the evaluation of the iron status [26], [27]. We examined various iron metabolism indicators in the workers' blood, and our results showed that BIC was an independent predictor of significantly lower blood lead levels in the multivariate analyses that were adjusted for age, sex, smoking, drinking, education, and lead exposure. The mean iron metabolism indexes were in the normal range for both lead exposed and unexposed workers. Evidence was found of an interaction between the *H63D* genotype and the body iron status.

Our current study also found that the *H63D* genotype has an interactive effect with BPb on Tf: higher BPb levels were associated with a lower Tf, and with an elevation in BPb, the Tf dropped more in workers with the *H63D* variant than with the subjects with the wild-type, although the R square for the multiple linear analysis that was adjusted for the confounders only to 0.141. It is known that Tf is a powerful chelator, capable of binding iron tightly but reversibly; a molecule of Tf can bind two atoms of ferric iron with high affinity, and a significant serum Tf decrease indicates that there may be a clinical iron overload in the subject's body [28]. Additionally, a study of twins in Jahanshad et al. [29] revealed that the *H63D* variant was associated with decreased transferrin levels and that AD patients who carry only the *H63D* heterozygous variant showed higher levels of iron and lower levels of transferrin than the wild-type AD patients [30]. A higher blood lead was also associated with the *HFE* mutation in young children (with 16.7% *H63D* heterozygous variant and 3.3% *C282Y* variant), with an elevation in the need for body iron [5]. Furthermore, Pb has been shown to bind directly to transferrin, down-regulating Tf gene expression [31]. Our results suggest that the *H63D* variant affects both lead metabolism and iron metabolism, and our investigation further enriches the knowledge of the interaction and relationship between Pb exposure and iron metabolism.

We tested for the effects of a number of covariants that have been reported to affect blood lead levels in other general population-based studies. There were no significant effects of smoking and alcohol use, which is different from previously reports, except for sex [6], [32]. The mean values of the blood lead were significantly higher than those reported elsewhere because subjects in our study were workers who were occupationally exposed to lead. The measures of environmental exposure (exposure duration and the type of work) show independent effects on lead concentrations.

The conclusions of this study are subject to several limitations. First, our sample was restricted to a homogeneous sample of Chinese workers who were occupationally exposed to lead, who displayed a lower prevalence of the allelic frequency of the *H63D* variant (2.98%) than people from Europe (12∼15%), the America (14∼15%), North Africa (13∼17%) and South Korea (3.8%) but displayed a higher prevalence than people from Japan (0.99%) [33], [34]. Furthermore, our sample contained no *C282Y* variant carriers. Although studies outside Asia have found that the *C282Y* variant is the most common variant genotype and is highly prevalent (50–100%) in *C282Y* homozygous variant [35], the frequency of the *H63D* variant is higher than the *C282Y* variant in Asia. The roles of the *H63D* and *C282Y* variants in producing hemochromatosis were believed to different [36]; with regard to the *H63D* variant, its effect on the body iron stores was much milder than the *C282Y* variant, particularly the heterozygous and iron overload was scarcely observed [37]. This finding is in line with our study within the normal range of iron metabolism indexes in our subjects. Therefore, it will be important to replicate this study in different occupationally lead-exposed populations. Second, we measured Pb only in the blood and urine. Although the BPb and UPb levels are the most common method for establishing the degree of exposure in humans, the factors described may affect either the lead absorption and body burden or only its distribution within the body. In the latter case, some people could have high blood lead levels but an acceptable total body lead content, or vice versa. Distinguishing between these possibilities would require the measurement of lead in the bones. Third, the control population was not engaged in lead operations in the same lead smelter, and their blood lead levels were higher than in the general population, even though there was no likelihood of a greater risk to lead exposure within the control population. It would be preferable to choose controls from the general population or use double controls, using subjects from both the lead smelter and the general population, to determine the association between lead exposure and iron metabolism.

## Conclusions

In summary, in a cross-sectional study of occupationally lead-exposed workers, the *H63D* genotype modified the association between lead exposure and iron metabolism with a positive association between the body iron content and blood lead in subjects carrying the *H63D* variant.
